# Embryonic blood-cerebrospinal fluid barrier formation and function

**DOI:** 10.3389/fnins.2014.00343

**Published:** 2014-10-28

**Authors:** David Bueno, Maryam Parvas, Ismaïl Hermelo, Jordi Garcia-Fernàndez

**Affiliations:** Department of Genetics, Faculty of Biological Sciences, University of BarcelonaBarcelona, Spain

**Keywords:** embryonic cerebrospinal fluid, blood-eCSF barrier, primary neurogenesis, neural progenitor cells, brain development, cephalic vesicles

## Abstract

During embryonic development and adult life, brain cavities and ventricles are filled with cerebrospinal fluid (CSF). CSF has attracted interest as an active signaling medium that regulates brain development, homeostasis and disease. CSF is a complex protein-rich fluid containing growth factors and signaling molecules that regulate multiple cell functions in the central nervous system (CNS). The composition and substance concentrations of CSF are tightly controlled. In recent years, it has been demonstrated that embryonic CSF (eCSF) has a key function as a fluid pathway for delivering diffusible signals to the developing brain, thus contributing to the proliferation, differentiation and survival of neural progenitor cells, and to the expansion and patterning of the brain. From fetal stages through to adult life, CSF is primarily produced by the choroid plexus. The development and functional activities of the choroid plexus and other blood–brain barrier (BBB) systems in adults and fetuses have been extensively analyzed. However, eCSF production and control of its homeostasis in embryos, from the closure of the anterior neuropore when the brain cavities become physiologically sealed, to the formation of the functional fetal choroid plexus, has not been studied in as much depth and remains open to debate. This review brings together the existing literature, some of which is based on experiments conducted by our research group, concerning the formation and function of a temporary embryonic blood–CSF barrier in the context of the crucial roles played by the molecules in eCSF.

## Introduction

Many civilizations have developed along riverbanks and seashores, in order to use the fluid medium to promote cohesion and transport, and to favor the survival of the people living at its edge. Surprisingly, the brain is also organized, from its embryonic beginnings and throughout adult life, around an extraordinarily dynamic and complex fluid, called cerebrospinal fluid (CSF), which the cells lining the brain and spinal cord bathe in. CSF is a subject that attracts growing interest in brain development research. In the last two decades, this has led to an understanding of normal adult brain function and disease, including migration of neuroblasts created in the subventricular zone of the adult brain from the lateral ventricles to the olfactory bulb (Sawamoto et al., 2006). CSF composition during embryogenesis and the fetal period is highly complex, as demonstrated by several proteomic analyses (Dziegielewska et al., 1980a, 1981; Fielitz et al., 1984; Gato et al., 2004; Parada et al., 2005a, 2006; Zappaterra et al., 2007). In addition, CSF composition and its biological properties change throughout development in a tightly regulated manner in parallel with its function, as demonstrated for some of the molecules contained within the embryonic CSF (eCSF), e.g., retinol binding protein (RBP), and fibroblast growth factor no. 2 (FGF2), with regard to the initiation of primary brain neurogenesis (Parvas et al., 2008a).

**Figure 1:**
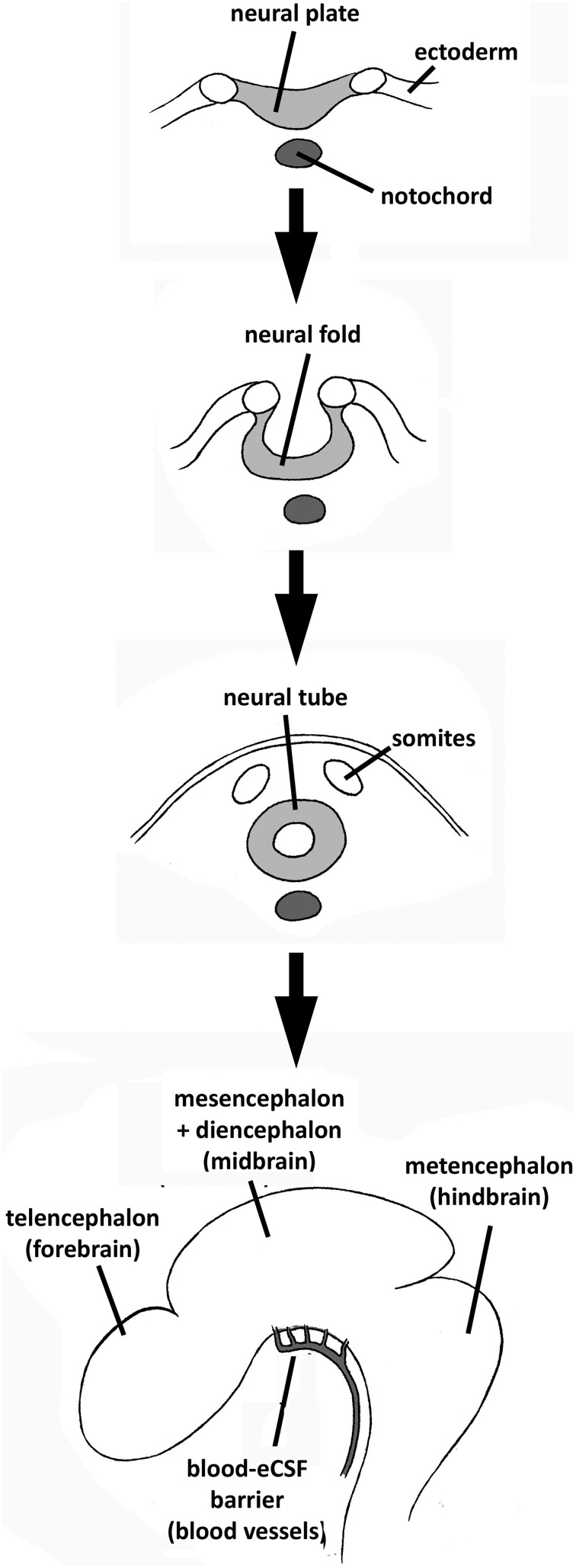
**Summary of CNS development and brain cavities formation from the differentiation of the neural plate to the initiation of brain primary neurogenesis (corresponding to embryonic stage E4 in chick embryos or E12.7 in rat embryos, according to the experiments reviewed in this paper)**. The embryonic blood–eCSF barrier is also indicated.

The development of the brain involves three main distinct phases: formation of the tube from the neural plate; polarization of the tube into a posterior spinal cord, and an anterior expanded brain, which is initially a hollow eCSF-filled vesicle surrounded by a pseudo-monostratified neuroepithelium; and histiogenesis of the neuroepithelium (Figure 1; for a general description of central nervous system (CNS) development, see Smith and Schoenwolf, 1997; Copp et al., 2003; Copp, 2005). During most of the last century, developmental neuroscience focused primarily on neuroepithelial events at the molecular, cellular, and tissue level. For example, it has been demonstrated that diffusible molecules such as growth factors and morphogens secreted locally by organizing centers regulate these processes by controlling neighboring cells in an autocrine/paracrine way (Bally-Cuif and Boncinelli, 1997), and that they regulate the expression of a number of transcription factors through which complex molecular, and genetic networks are established.

Only in the last two decades has the eCSF in the cavities of the brain primordium started to attract attention and be considered an essential part of the developing brain which exerts a direct influence on basic neuroepithelial cell behavior, i.e., promoting cellular survival, replication, and neurogenic differentiation, and also contributing to the regulation of neuroepithelial gene expression, thus playing a key role in brain development (Gato et al., 2005; Martin et al., 2006, 2009; Parada et al., 2008a; Alonso et al., 2011; Feliciano et al., 2014). In fetuses and adults, these cavities become the brain's ventricular system, since eCSF is the precursor of fetal and adult CSF (reviewed by Miyan et al., 2003). It has also been demonstrated that fetal CSF (fCSF) contributes to cortical development (Miyan et al., 2006).

**Figure 2:**
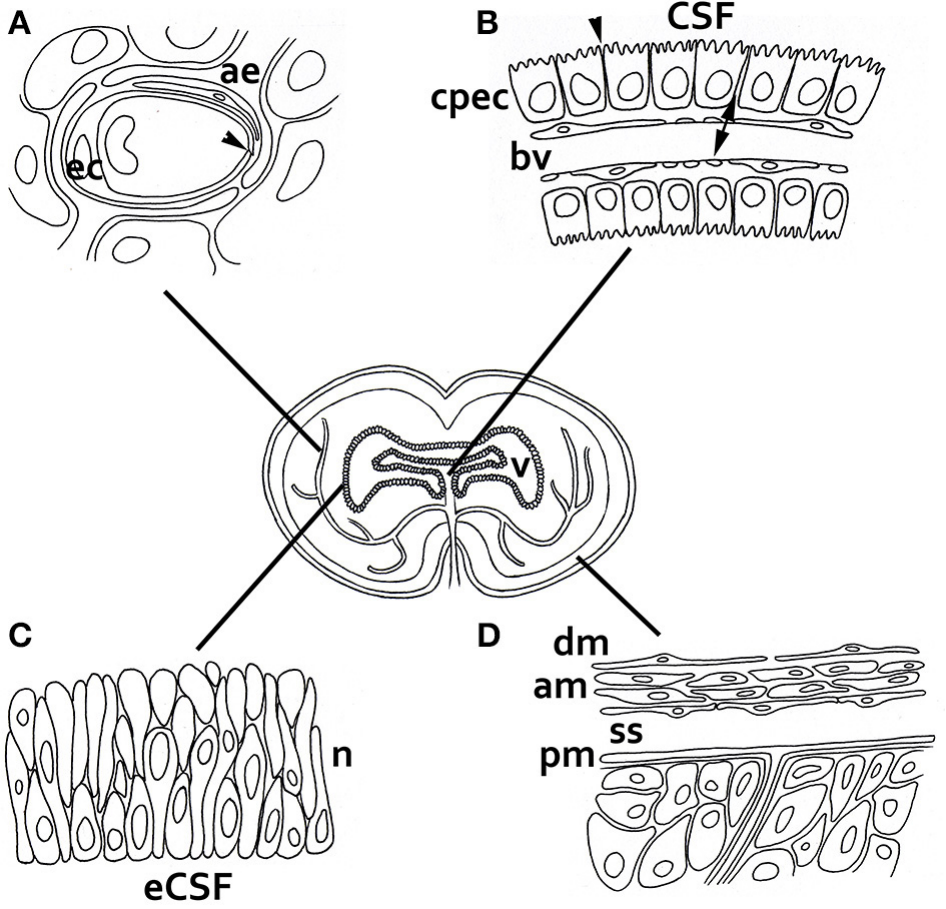
**Barriers of the developing and adult brain. (A)** The blood–brain barrier between the lumen of cerebral blood vessels and the brain parenchyma. The endothelial cells have luminal tight junctions (arrowheads) that form a physical barrier, preventing the movement of molecules from the blood plasma, except those that are allowed to enter the brain via active and specific transport. In embryos, some blood vessels and the neuroectodermal cells adjacent to them also perform this function. **(B)** The blood–CSF barrier between choroid plexus blood vessels and the adult and fetal CSF. These blood vessels are fenestrated and form a non-restrictive barrier (arrows), but the choroid plexus epithelial cells are joined by tight junctions to stop the movement of molecules (arrowheads). **(C)** The inner CSF–brain barrier between the eCSF and the brain parenchyma, which is present only during early development. The neuroependymal cells lining the brain cavities do not allow the exchange of large molecules such as proteins between the eCSF and brain, but they do allow exchange of smaller molecules such as sucrose. This implies that the influence of eCSF on basic neuroepithelial cell behavior—e.g., promoting cellular survival, replication and neurogenic differentiation—has to be accounted for through specific receptors located at the apical pole of neural progenitor cells, which are in contact with the lumen of the cavities. There is no crossing restriction in the adult brain. **(D)** The outer CSF–brain barrier between the subarachnoid space and overlaying structures. The blood vessels are fenestrated, but the outer cells of the arachnoid membrane are connected by tight junctions. Abbreviations: ae, astrocitic endfoot; am, arachnoid membrane; bv, blood vessel; cpec, choroid plexus endothelial cell; dm, dura mater; ec, endothelial cell; n, neuroepithelium; pm, pia mater; ss, subarachnoid space; v, ventricle.

CSF composition and homeostasis in fetuses and the adult brain are tightly regulated by the choroid plexus, whose epithelial cells establish a blood–CSF barrier (Figure 2; Møllgård et al., 1979; Tauc et al., 1984). The choroid plexus is a vascular structure in the brain ventricles that manufactures CSF by promoting the transport of certain molecules from blood plasma and producing others that are delivered directly to the ventricles. Acting in parallel, CNS homeostasis in adult vertebrates is also controlled by the blood–brain barrier (BBB) vessels in the brain; this significantly impedes entry from the blood to the brain of virtually all molecules, except those that are small and lipophilic. However, BBB vessels also allow sets of small and large hydrophilic molecules, e.g., gene products, to enter the brain via active transport (reviewed by Rubin and Staddon, 1999; see Figure 2 for a more extensive summary of brain barriers).

In parallel with eCSF studies, control of eCSF production and homeostasis in vertebrate embryos from the closure of the anterior neuropore to the formation of the choroid plexus during fetal stages have not been analyzed until recently. However, this developmental stage is particularly interesting for brain formation, since it is mainly characterized by rapid brain anlagen growth and initiation of primary neurogenesis in the neural progenitor cells lining the cavities. This review brings together the existing literature on the formation and function of embryonic blood–CSF barriers, from the closure of the anterior neuropore to the formation of choroid plexuses in vertebrates, using avians (chicks), and mammals (mainly mice and rats) as model systems. We highlight the crucial role played by eCSF in neural development. As stated by Gato (2013) in a short but revealing commentary, “eCSF—and by extension the barriers and mechanisms controlling its formation, composition and homeostasis—constitute a rising subject in brain development.”

## Phylogenetic origin of the CSF system

**Figure 3:**
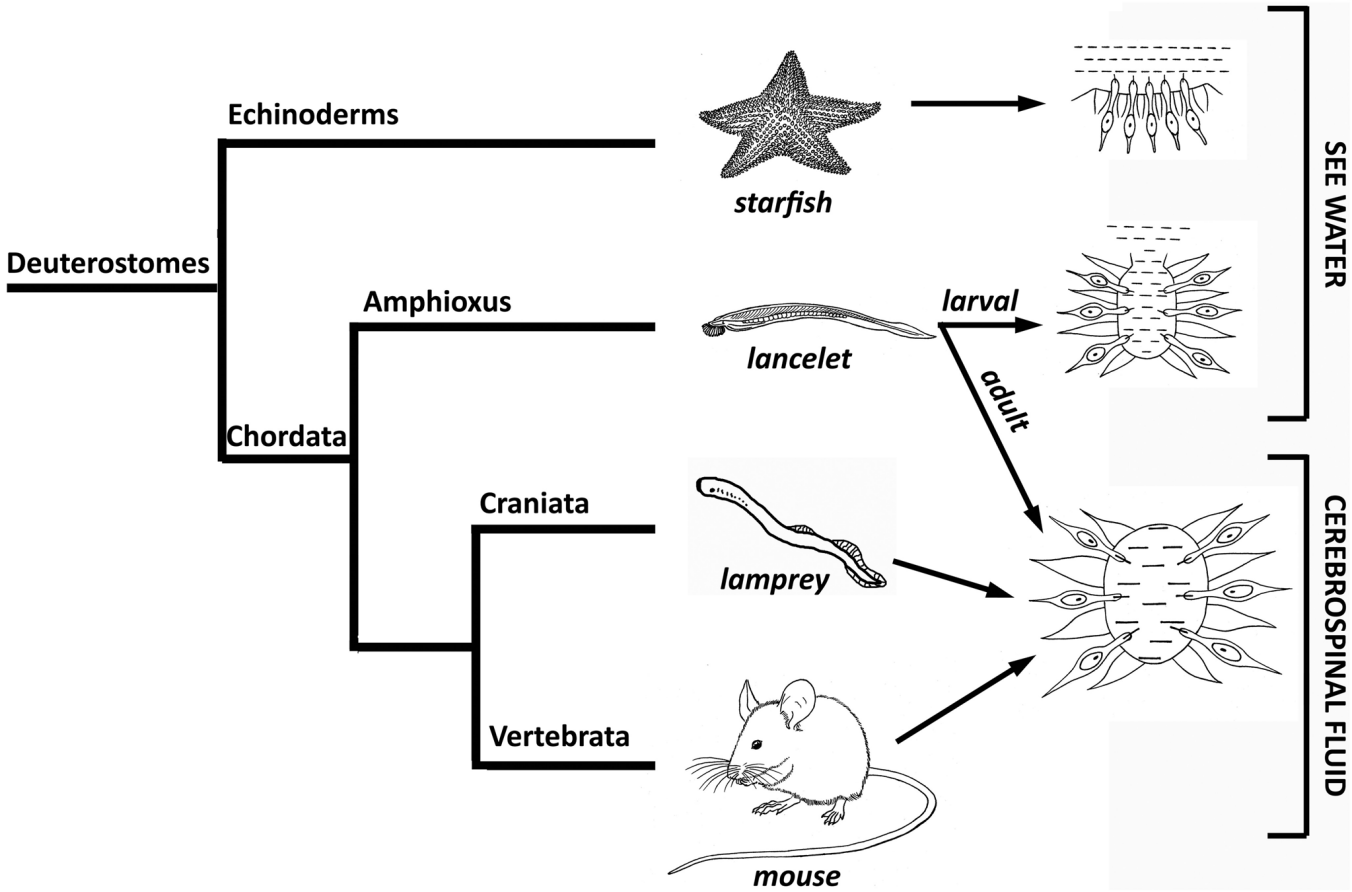
**Scheme of the evolution of the phylogenetic increase in the control that the chemical environment has over the cerebrospinal fluid**.

It is thought that the CSF system evolved in the deuterostome lineage from the ancestor of echinoderms and chordates to maintain the chemical environment necessary for the functioning of the cells of the CNS, including the neuroendocrine pathways (Brocklehurst, 1979). For example, it is known that in starfish, the neurons of the radial nerves and “brain” are always in contact with seawater; this allows them to detect the composition of the seawater, as well as other physical parameters such as water temperature, and modulate the activity of the nervous system accordingly. However, its open nature means that this system does not respond to neuroendocrine or other signaling molecule pathways (Figure 3).

During the larval stages of morphologically and evolutionarily more complex lancelets, such as the chordate amphioxus *Branchiostoma lanceolatum*, seawater enters the ventricular lumen of the “brain” through the anterior neuropore, which remains open until adulthood. Seawater represents the first “internal” fluid environment of lancelet brain tissue and depends directly on the environment. However, when lancelets reach adulthood, the anterior neuropore closes and the ventricular lumen of the “brain” becomes a closed system (Figure 3). Thus, the composition of this fluid no longer depends directly on the environment, but on the activity and metabolism of the organism. Finally, the composition of eCSF in craniata, such as lampreys, and vertebrates depends exclusively on the surrounding tissue and the rest of the embryo from early embryonic stages, since the anterior neuropore closes very early in neural tube development, before brain primary neurogenesis starts. This suggests that this condition was first developed by the ancestor of these two phylogenetic groups (Figure 3; reviewed by Vígh et al., 2004).

## eCSF protein content

In vertebrates, brain development depends on regional character specification, which in turn depends on a progressive series of precisely coordinated morphogenetic movements in combination with cell proliferation and differentiation. These processes are controlled by the sequential action of patterning molecules, which give neural progenitor cells specific characteristics, including transcription factors and diffusible molecules such as growth factors and morphogens (Martinez, 2001; Stern, 2001, 2002). During early stages of brain development, when the architecture of the brain anlagen is formed by a pseudo-monostratified neuroepithelium that develops around the cephalic cavities with its lining surface in close contact with the eCSF, these diffusible molecules may come from both the surrounding tissues, such as the mesencephalic-rhombencephalic isthmus (IsO) organizing center (Nakamura et al., 2005), and eCSF, which may be used as a fluid pathway for their delivery.

Classic studies performed on chick embryos at developmental stages E2.5 to E6.5 (E = embryonic developmental day) showed that eCSF has a higher concentration of total proteins than adult CSF (Birge et al., 1974; Gato et al., 2004). A similar situation has been observed in sheep at E35 (youngest fetus) as compared with E60 (sheep gestation, 145 days), and in rat from E12 to E22 (birth), and neonatal. Some phylogenetic differences in CSF maturation have been identified, however, when comparing these mammalian species, regarding, for example, the developmental stages at which the reduction in total CSF protein occurs (Dziegielewska et al., 1980a,b; 1981; Checiu et al., 1984; Fielitz et al., 1984).

The first published studies of the specific protein content of eCSF were based on crossed immunoelectrophoresis to identify individual proteins, and radial immunodiffusion to quantitate them. It was suggested that the protein fractions identified in sheep eCSF were albumin, fetuin, alpha-fetoprotein, transferrin, lipoproteins, alpha1-antitypsin, and prealbumin (Dziegielewska et al., 1980a, 1981). Rat eCSF exhibits a similar electrophoretic pattern, with the possible presence of IgGs (Dziegielewska et al., 1981). As regards chick eCSF, Gato et al. (2004) identified 21 protein fractions by SDS-PAGE protein separation and molecular mass inference, which coincide with previously reported proteins such as transferrin, alpha-fetoprotein, immunoglobulins, and transthyretin.

Proteomic analyses of avian and mammalian eCSF that confirm and significantly expand on most of the abovementioned findings have also been published. The first reports to use conventional proteomic techniques (2D-electrophoresis, in-gel digestion, and ESI-MS/MS mass spectrometry analysis) on chick and rat eCSF at E4 and E12.5, respectively, which correspond to the developmental stages in which brain development is characterized by maximum neuroepithelial progenitor cell proliferation and the beginning of neural differentiation, showed the presence of dozens of different specific proteins within this fluid, most of which may be involved in brain development (Parada et al., 2005a, 2006). These proteins were classified into several different groups according to their functional characteristics, as previously reported in systems other than eCSF (Table 1). The most remarkable dissimilarity between chick and rat eCSF proteomes is the presence of enzymes and enzyme regulators in rat eCSF, as well as an increased number of members of the apolipoprotein family, from two different apolipoproteins identified in chicks, AI and AIV, to at least five in rats, including AI and AIV, as well as B, E, and M. It has been suggested that this relates to the higher complexity of the CNS in mammals, although apolipoprotein B was later identified in chick eCSF by Western-blot analysis using specific antibodies (Parada et al., 2008a). These dissimilarities may also be due to differences in choroid plexus formation. E4.5 chick embryos correspond to a developmental stage 2.5 days before the first appearance of the choroid plexus; while in E12.5 rat embryos, transthyretin-positive choroid plexus primordium starts to be detected. However, rat choroid plexus primordium at this developmental stage is not fully functional and, as discussed below (see Sections Protein Traffic Occurs in Very Specific Blood Vessels and Includes Both Endothelial and Neuroepithelial Cells, and Traffic of Water, ions, And Glucose Occurs at the Same Transport Site as Proteins), these rat embryos still maintain a specific and temporary embryonic blood–eCSF barrier.

**Table 1:** **Summary of the classifications of proteins identified in chick, rat and human eCSF proteomes**.

**Chick eCSF**	**Rat eCSF**	**Human eCSF**
Parada et al., 2006	Parada et al., 2005a	Zappaterra et al., 2007
Bueno et al., unpublished results Extracellular matrix Osmotic pressure and ion carriers Cell death and quiescence Apolipoproteins Retinol and vitamin D carriers Antioxidant and antimicrobial Intracellular Unknown	Extracellular matrix Osmotic pressure and ion carriers Regulators of lipid metabolism Retinol and corticosteroid carriers Enzymes and enzyme regulators Antioxidant and antimicrobial Unknown function during embryonic development	According to subcellular location Secreted Secreted, extracellular matrix Cell membrane Cytoplasm Nucleus Intracellular Golgi Lysosome
	————————	
	Zappaterra et al., 2007	
	Bueno et al., unpublished results	
	According to subcellular location Secreted Secreted, extracellular matrix Cell membrane Cytoplasm Nucleus Intracellular Golgi Lysosome ER	According to molecular function Cell adhesion Chaperone Cytoskeletal Defense/immunity Extracellular matrix Hydrolase Kinase Ligase Membrane traffic Miscellanea Unclassified Nucleic acid binding Oxidoreductase Phosphatase Protease Receptor Calcium binding Regulatory molecule Signaling molecule Synthase and synthetase Transcription factor Transfer Transferase Transporter Cell junction protein
	According to molecular function Cell adhesion Chaperone Cytoskeletal Defense/immunity Extracellular matrix Hydrolase Kinase Ligase Membrane traffic Miscellanea Unclassified Nucleic acid binding Oxidoreductase Phosphatase Protease Receptor Calcium binding Regulatory molecule Signaling molecule Synthase and synthetase Transcription factor Transfer Transferase Transporter Isomerase	According to biological process 1. Neuronal activities 2. Signal transduction 3. Developmental processes 4. Cell proliferation and differentiation 5. Coenzyme and prosthetic group metabolism 6. Cell structure and motility 7. Immunity and defense 8. Apoptosis 9. Oncogenesis 10. Muscle contraction 11. Transport 12. Blood circulation and gas exchange 13. Carbohydrate metabolism 14. Nucleoside, nucleotide and nucleic acid metabolism 15. Homeostasis 16. Protein metabolism and modification 17. Cell cycle 18. Intracellular protein traffic 18. Cell adhesion 19. Lipid, fatty acid and steroid metabolism 20. Sensory perception 21. Electron transport 22. Amino acid metabolism 23. Biological process unclassified 24. Protein targeting and location 25. Miscellaneous
	According to biological process 1. Neuronal activities 2. Signal transduction 3. Developmental processes 4. Cell proliferation and differentiation 5. Coenzyme and prosthetic group metabolism 6. Cell structure and motility 7. Immunity and defense 8. Apoptosis 9. Oncogenesis 10. Muscle contraction 11. Transport 12. Blood circulation and gas exchange 13. Carbohydrate metabolism 14. Nucleoside, nucleotide and nucleic acid metabolism 15. Homeostasis 16. Protein metabolism and modification 17. Cell cycle 18. Intracellular protein traffic 18. Cell adhesion 19. Lipid, fatty acid and steroid metabolism 20. Sensory perception 21. Electron transport 22. Amino acid metabolism 23. Biological process unclassified 24. Protein targeting and location 25. Miscellaneous	

*References are shown in the table*.

Zappaterra et al. (2007) also reported an extensive and thorough proteome analysis of rat eCSF from three different stages: E12.5, E14.5 from both the lateral and fourth ventricles, and E17.5 during cortical development. They identified 423, 318, 249, and 382 proteins, respectively, which were also classified into several groups according to their function (Table 1). The human eCSF proteome has been analyzed at Carnegie Stage (CG) 20 (Zappaterra et al., 2007), revealing the presence of 188 proteins; 130 of which are shared with rats. The categorization of these proteins based on their molecular function and the biological processes they are involved in are almost identical, which would suggest that they represent essential eCSF functions.

More recently, proteomic analysis performed on both eCSF from chick and rat embryos at E4 and E13 respectively, but using a more sensitive technique (a high-performance liquid chromatography ion-trap column coupled to an ESI MS/MS mass spectrometer), showed the presence of 121 (chick), and 100 (rat) different proteins within the fluid, although all of them may be classified in the same general groups (Bueno et al., unpublished results). All of these studies, whose overall results are relatively similar, have proved to be complementary and reinforce the putative capacity of eCSF to influence the behavior of neuroepithelial cells through the molecules it contains, as has been demonstrated in several studies (Gato et al., 2005; Martin et al., 2006, 2009; Parada et al., 2008a,b).

However, one of the limitations of this proteomic analysis is the almost complete lack of growth factors and cytokines within eCSF proteomes, since these molecules, which are crucial for controlling developmental processes, exert their actions at very low concentrations. They may therefore be masked by the more abundant proteins or may be below the detection threshold of the techniques used. Nevertheless, Western-blot analysis of both avian and mammalian eCSF using specific antibodies revealed the presence of some growth factors and cytokines within this fluid. These include fibroblast growth factor 2 (FGF2) (Martin et al., 2006), epidermal growth factor (EGF) (Birecree et al., 1991), and leukemia inhibitory factor (Hatta et al., 2006), which are known to be involved in the regulation of a number of developmental processes.

Although interpreting these different results is complex because of the different classifications used (see Table 1), and the fact that some gene products may correspond to different functional groups in different studies, it is evident that eCSF acts as a fluid pathway to transfer proteins to and from the neuroepithelial cells. It thus influences neuroepithelial cell behavior and, hence, brain development (Gato et al., 2005; Parada et al., 2005a, 2008a,b; Martin et al., 2006, 2009; Alonso et al., 2011; Castells et al., 2012; see Gato and Desmond, 2009 for a review).

## eCSF function

Through the specific molecules it contains and certain of its mechanical properties, eCSF plays several crucial roles during the early stages of brain development: just after the closure of the anterior neuropore when the brain ventricles become physiologically sealed cavities, during initial brain anlagen growth, and during the initiation of the primary neurogenesis of the neural progenitor cells. Using several experimental approaches in chick and mouse embryos, it has been reported that the progressive increase in eCSF volume exerts positive pressure against the neuroepithelial walls and generates an expansive force, thus contributing to brain expansion. An experimentally inflicted decrease in eCSF pressure leads to severe dysmorphogenesis and brain collapse (Desmond and Jacobson, 1977; Desmond and Levitan, 2002). The rapid expansion, which accounts for 70% of brain enlargement in the early stages, occurs in parallel with occlusion of the spinal neurocoele (Desmond and Jacobson, 1977). This renders the cephalic cavities a physiologically sealed system, which would indicate the existence of mechanisms that control the influx of water and ions into the brain cavities. Moreover, as proteoglycans are the major components of the extracellular matrix in embryo brain cavities (Alonso et al., 1998, 1999), it has been suggested that the special osmotic properties of chondroitin sulfate proteoglycan and other proteoglycans in eCSF may cause water retention in the cavities. This would generate and regulate the inner cephalic hydrostatic pressure, although other proteins, e.g., major eCSF proteins, may also contribute to this function.

In addition to the mechanical properties of eCSF, it has also been shown to contribute to the regulation of some basic cellular functions, e.g., the survival, proliferation, and differentiation of neural progenitor cells. Through the use of *in vitro* cultures of mesencephalic neuroectodermal explants from both chick and rat embryos at E4 and E12.5, respectively, the diffusible molecules in eCSF have been shown to actively contribute to the regulation of the survival, proliferation, and neurogenesis of neuroepithelial progenitor cells, directly from this embryonic fluid (Gato et al., 2005; Martin et al., 2009). Mesencephalic explants cultured with only a chemically defined medium show a reduction in the number of proliferating cells, an increased rate of apoptotic cells, and a severe decrease in the number of cells engaged in the process of neural differentiation (i.e., primary neurogenesis) compared to control embryos. However, when the explants are cultured with a chemically defined medium supplemented with eCSF, the cellular parameters remain close to those of the control embryos.

Most of the proteins identified in eCSF in the abovementioned proteomic analysis have known physiological functions during embryonic development that are consistent with the overall roles reported for eCSF during CNS development. Functional *in vivo* and *in vitro* analysis of the particular role of some of these gene products at the beginning of primary neurogenesis, before the formation of functional choroid plexuses, has revealed their specific roles in the overall function of eCSF in neuroepithelial progenitor cell behavior. For example, it has been reported that the deglycosylation of eCSF proteoglycans with beta-d-xyloside alters brain enlargement in chick embryos (Alonso et al., 1998), and that immunoblocking of the FGF2 in eCSF severely disrupts neuroepithelial stem cell proliferation and differentiation (Martin et al., 2006). Similarly, it has been reported that the LDL lipid fraction, transported by apolipoprotein B in eCSF, is also involved in regulating neuroepithelial progenitor cell proliferation and differentiation (Parada et al., 2008a). Other reports have shown that retinol-binding protein (RBP) is responsible for transporting and transferring all*-trans*-retinol from the embryonic plasma to the eCSF, from where it reaches the neuroepithelium and is transformed into retinoic acid, the actual morphogen, by retinoic acid-synthesizing enzymes expressed in the neuroepithelium (Parada et al., 2008b; Alonso et al., 2011). Also that large glycoprotein SCO-spondin synthesized by the by the diencephalic roof plate contributes to the control of neuroepithelial cell proliferation via eCSF (Vera et al., 2013). As well as that eCSF nanovesicles carry evolutionarily conserved molecules that promote neural stem cell amplification (Feliciano et al., 2014); among other findings.

In addition to the effects of eCSF on neural progenitor cell behavior through some of the specific molecules it contains, its involvement in regulating the expression of genes known to be involved in brain patterning has also been established (Parada et al., 2005b). When dorsal mesencephalic neuroepithelial explants lacking IsO (a known organizing center for mesencephalon and brain development) or dorsal mesencephalic neuroepithelial explants including IsO are cultured in a chemically defined medium but in the absence of eCSF, the typical expression of dorsal mesencephalic, and IsO genes, i.e., *fgf8* and *otx2*, is disrupted. Conversely, when dorsal mesencephalic explants including IsO are cultured in an eCSF-supplemented medium, they do exhibit this expression. Interestingly, however, when dorsal mesencephalic explants lacking IsO are cultured with an eCSF-supplemented medium, they also show ectopic expression domains of *Shh* in the mesencephalic neuroectoderm; *Shh* is a gene that is typically expressed in the ventral but not dorsal neuroectoderm. Only the concurrence of eCSF within the culture medium and IsO in the dorsal mesencephalic explant makes this tissue mimic its typical pattern of gene expression, which would suggest that IsO and some molecules in eCSF exert a synergistic action.

## Mechanisms controlling eCSF formation and homeostasis

The crucial roles played by eCSF during initial brain development together with the complexity of its composition (it includes proteins that may account for the overall function of this fluid in neuroepithelial development) raise some interesting questions about the origin of the gene products and other molecules within it. Further questions concern how the homeostasis of this fluid is controlled at the beginning of primary neurogenesis, just after the closure of the anterior neuropore when the cephalic cavities become physiologically sealed, since the development of a functional choroid plexus is known to occur a bit later in development.

### The need for a blood–eCSF barrier function: a historical perspective

Developing choroid plexuses are first detected at E7 in chicks, at E12.5–E13 in rats and mice, and during the seventh week of gestation in humans (Bellairs and Osmond, 2005; Emerich et al., 2005). Despite the large amount of accurate information available on brain barriers encompassing fetal stages through to adulthood (Ferguson and Woodbury, 1969; Parada et al., 2007; Johanson et al., 2008, 2011; Gato and Desmond, 2009; Liddelow, 2011; Ek et al., 2012; Zappaterra and Lehtinen, 2012), the existence of an embryonic blood–eCSF barrier that controls eCSF composition and homeostasis during early embryonic development remains in doubt. This is partially due to a common belief that barriers in the developing brain are immature, although this implies neither the absence of mechanisms that control eCSF composition and homeostasis, nor that the embryonic brain does not require an environment of great chemical stability.

A further complicating factor is the way in which blood–CSF barrier permeability has traditionally been interpreted. Conventionally, the fact that the ratio of the concentration of particular molecules in CSF with respect to that in blood plasma is much higher in the developing brain than in the adult brain (Ferguson and Woodbury, 1969; Dziegielewska et al., 1979; Habgood et al., 1993; Ek et al., 2001) has been interpreted as evidence of greater barrier permeability (Spector and Johanson, 1989; Kniesel et al., 1996; Engelhardt, 2003; Lee et al., 2003). However, the morphological basis of these barriers formed by means of tight junctions is present from the earliest stages of development between endothelial blood vessel cells in BBB areas, as well as in the epithelial cells of the choroid plexuses in the blood–CSF barrier (Møllgård et al., 1979; Saunders and Møllgård, 1984; Tauc et al., 1984). To reconcile these two sets of evidence, it has been suggested that different transcellular mechanisms operate for protein and small molecule transfer across the embryonic blood–CSF interface (Johansson et al., 2006).

In the past 8 years, a number of studies have provided increasing evidence of the existence of a transient blood–eCSF barrier mechanism in both avian and mammal embryos between the closure of the anterior neuropore and the start of choroid plexus functionality (see below). It has also been demonstrated that at this developmental stage in avians, major eCSF protein fractions are not synthesized by the neuroectodermal cells, but are synthesized in other embryonic tissues or derived from the egg, and subsequently transported from the blood plasma to the cephalic cavities, most probably in a selective manner (Parvas et al., 2008b). These data suggest the need for a physiological blood–eCSF barrier to control the initial composition and homeostasis of the eCSF before the formation of a functional fetal choroid plexus.

### The cephalic neuroectoderm becomes impermeable just after closure of the anterior neuropore

The impermeability of the cephalic neuroectoderm is one of the key factors in the formation of a physiologically sealed system of brain cavities. Early experiments to test the integrity of the embryonic neuroectoderm relied on injected dyes such as trypan, or Evans blue, or enzymes such as horseradish peroxidase (HRP). They produced conflicting results, probably because of the fragility of embryos (Habgood et al., 1992). For example, Wakai and Hirokawa (1978) showed that blood–CSF interface permeability to HRP may follow a free diffusion pattern beginning to decrease at E12–E14 in chick embryos; although their results are probably an artifact due to the very high concentrations of HRP used in the work. However, much more recent experiments in which small concentrations of FGF2 conjugated to FITC or alternatively other proteins of a similar size not normally present in the eCSF are injected into the outflow of the embryo heart, show the existence of selective transport from the blood plasma to the eCSF from E4 onwards (Martin et al., 2006; Parvas et al., 2008a).

**Figure 4:**
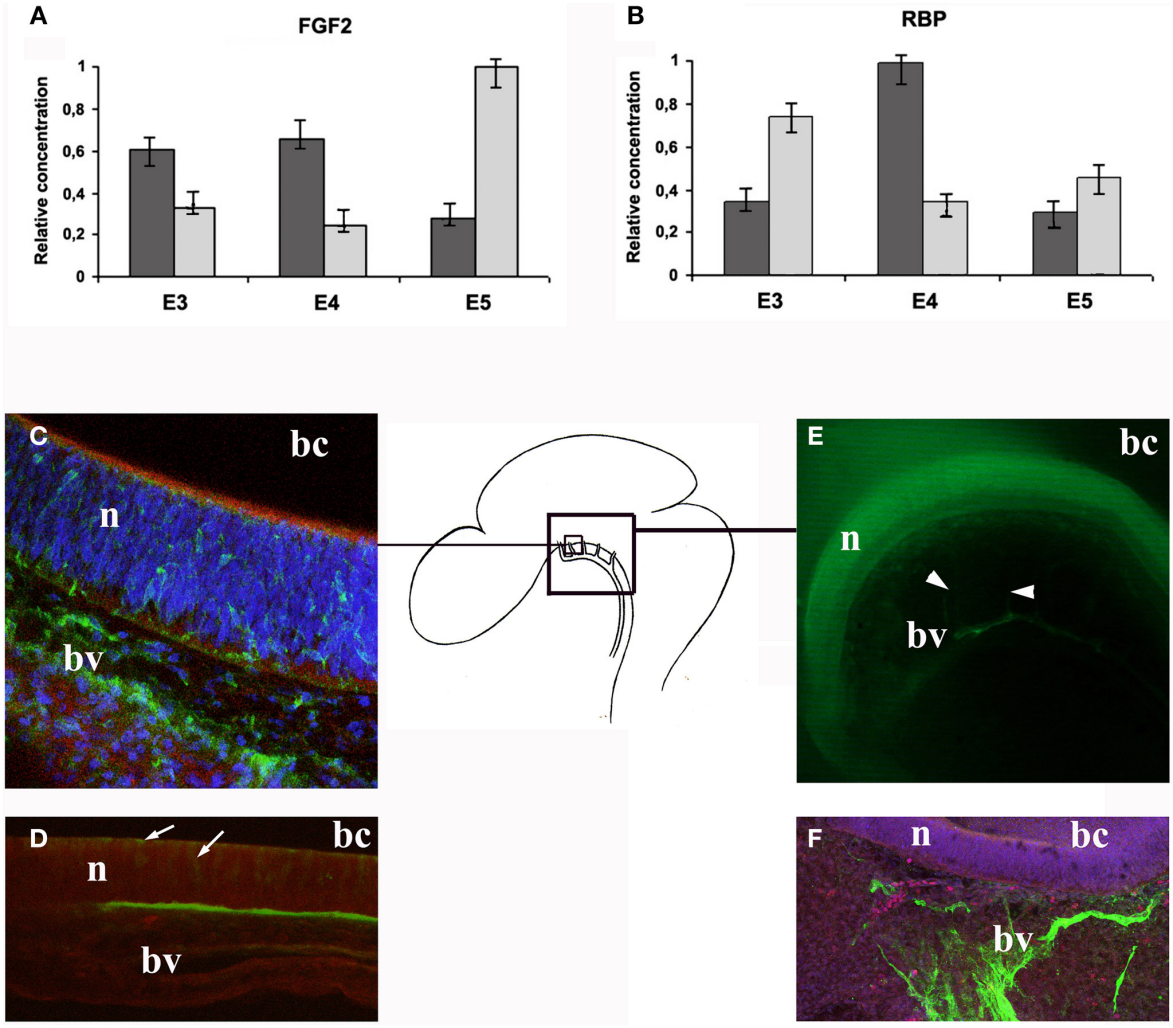
**Transport of molecules across the blood–eCSF barrier in chick embryos. (A-B)** Relative concentrations of some endogenous proteins in the embryonic serum (light gray), and in the eCSF (dark gray), at E3, E4, and E5 developmental stages (Parvas et al., 2008a). **(C)** Vibratome section showing transport of BDA3000 injected into the cephalic cavities at the ventral mesencephalon, lateral to the floor plate, at E4 (Parvas et al., 2008a). Note the presence of fluorescent dextran in both blood vessels (bv) and across the neuroectoderm (n). **(D)** Vibratome section showing transport of BSA microinjected into the cephalic cavities from the eCSF to the embryonic serum, at E4 (Parvas et al., 2008a). Note the accumulation of BSA in the mesenchyme, at the interface between the neuroectoderm (n) and the blood vessels (bv). Also note the presence of BSA within neuroectodermal cells (arrows). **(E)** AQP1 immunostaining of half head, at E5. Note the immunostained blood vessels and capillary sprouts (arrowheads) (Parvas and Bueno, 2010b). **(F)** Magnification of a blood vessel immunostained with an anti-AQP1 antibody, at E6. Abbreviations: bc, brain cavity; bv, brain vesicles; n, neuroectoderm.

In a similar way, the microinjection of a small-sized tracer, biotin dextran amine of 3 kDa (BDA3000), into the outflow of the heart or the cephalic cavity of embryos at E3 and E4, showed that from E4 onwards its transport is restricted to a small subset of endothelial cells and cells adjacent to neuroepithelial cells, located in the ventral mesencephalon and the most anterior part of the ventral prosencephalon, lateral to the floor plate, via transcellular routes (Parvas et al., 2008a) (Figure 4). The existence of these specialist transport cells is a key factor in blood–eCSF barrier activity and in some respects may parallel the function of the fetal choroid plexus (Møllgård and Saunders, 1977; Balslev et al., 1997; Ek et al., 2001; Johansson et al., 2006).

### Protein traffic is specific and homeostasis tightly regulated

Initially, eCSF is derived from trapped amniotic fluid; a situation formally equivalent to that of lancelets with respect to seawater during the shift from larval to adult life (see Section Phylogenetic Origin of the CSF System Above; Vígh et al., 2004). After the closure of the anterior neuropore, however, the brain cavities become a physiologically sealed system, hence its composition becomes tightly regulated. Interestingly, most of the molecules identified in chick eCSF are not produced by the neuroectoderm itself. They are stored in the yolk or white of the egg and taken up by the chorioallantoic membrane, or produced by other embryonic structures. This would suggest that they are transported from the production or storage site to the eCSF, most probably via the blood plasma (Martin et al., 2006; Parvas et al., 2008a,b).

In this way, the quantification of the relative concentration of endogenous chick proteins normally present in both blood plasma and the eCSF, e.g., ovalbumin, pRBP, and FGF2, at different developmental stages, demonstrated that their physiological ratio is developmentally regulated. They vary from E3 to E5 in a way which is consistent with their known activities in brain development (Parvas et al., 2008a; Figure 4). For example, the eCSF/blood plasma ratio for FGF2 is higher at E5 than at E3 or E4, the developmental period at which this growth factor acts from the eCSF to influence neuroepithelial progenitor cell proliferation (Martin et al., 2006). While for pRBP, the eCSF/blood plasma ratio is much higher at E4 than at E3 or E5, coinciding exactly with its reported period of activity in retinol transport from blood plasma to the eCSF (Parada et al., 2008b).

It has also been demonstrated that the transfer of microinjected proteins across the blood–eCSF interface is protein-specific (Parvas et al., 2008a). Thus, proteins from non-chick sources are never transported from the blood plasma to the eCSF when experimentally microinjected into the outflow of the heart; with the notable exception of HRP, as discussed above. In the same way, neither were proteins which are not normally present in eCSF but are in the blood plasma transported from the blood plasma to this fluid, even when their concentration was experimentally increased 10- to 100-fold by microinjection (Parvas et al., 2008a).

Conversely, when chick proteins such as ovalbumin, FGF2 and pRBP, which are transported between these two embryonic compartments under normal physiological conditions, were bound to FITC to distinguish them from their endogenously produced counterparts and experimentally microinjected into the outflow of the heart, they were actively transported across the blood–eCSF interface (Parvas et al., 2008a; Figure 4). However, the transfer ratio of these proteins was maintained in its normal range, indicating that eCSF homeostasis is strongly controlled by the barrier system. For example, when ovalbumin, FGF2 or pRBP bound to FITC were microinjected into the outflow of the heart of chick embryos at E4, they were effectively transferred from the blood plasma to the eCSF, but the total concentration of each of these molecules did not increase in eCSF: it remained stable despite their 10- to 100-fold increase in the blood plasma (Parvas et al., 2008a). In other words, the rate of traffic does not depend on the relative concentrations between the blood plasma and the eCSF, but on some kind of “sensor” that controls eCSF homeostasis, paralleling those identified in the choroid plexus for several sets of molecules.

Moreover, the experimental microinjection of these chick endogenous proteins into the cephalic cavities to directly raise their concentration in the eCSF was rapidly compensated by the eCSF–blood plasma transport system (Parvas et al., 2008a). Similarly, when the microinjected proteins came from non-chick sources, they were rapidly eliminated from the eCSF (Figure 4). All of these data strongly suggest blood–eCSF barrier activity in both directions (from blood plasma to eCSF and vice versa) to control eCSF composition before the formation of the functional fetal choroid plexus, thereby ensuring that the eCSF activity is maximally efficient during neural development (Parvas and Bueno, 2011; Castells et al., 2012).

The importance of the blood–eCSF barrier in the control of eCSF composition and homeostasis, and thus, indirectly, in early brain development, has been tested by disrupting it with 6-aminonicotinamide gliotoxin (6-AN); a known antimetabolite of nicotinamide that blocks protein transport across the BBB by halting transcellular caveolae transport (Bertossi et al., 2003; Parvas and Bueno, 2011). Chick embryos treated with 6-AN showed caveolae transport disruption and a lack of protein transport across the blood–eCSF barrier; and this had a clear effect on neuroepithelial cell survival, proliferation and neurogenesis. This blockage also disrupted water influx to the eCSF, leading to an alteration in brain anlagen growth.

### Protein traffic occurs in very specific blood vessels and includes both endothelial and neuroepithelial cells

Histological analysis was carried out on embryos when transporting endogenous proteins linked to FITC microinjected into the outflow of the heart from the blood plasma to the eCSF, or when microinjected into the cephalic cavities from the eCSF to the blood plasma, as well as proteins from non-chick sources in the same direction. The results demonstrated that, within the developmental period analyzed, transfer from blood plasma to the eCSF, and vice versa occurs only in a specific area of the embryo: in the brain stem lateral to the floor plate, in the ventral mesencephalon and the most anterior part of the ventral prosencephalon (Figure 4). Interestingly, this location does not correspond to the area in which fetal choroid plexus development starts, i.e., from an invagination of the dorsal roof plate along the midline of the neural tube. This would seem to indicate that barrier properties are controlled by different independent systems at different stages of development. Moreover, the histological analysis showed that this blood–eCSF barrier function includes endothelial cells of specific perineural blood vessels and some vascular sprouts located within the neuroectoderm cells and adjacent neuroepithelial cells, as well as their adjacent neuroectodermal cells (Parvas et al., 2008a).

Similarly, immunohistological analysis revealed the presence of caveolin 1 in the endothelial cells of blood vessels involved in the blood–eCSF barrier function and in the adjacent neuroectodermal cells, which would indicate that proteins are transported via transcellular routes (Parvas and Bueno, 2010a). Caveolin 1 is the main structural component of caveolae, which are 50 to 100 nm vesicular invaginations of the plasma membrane that are involved in transcellular molecular transport, cell adhesion and signal transduction. Endothelial cells usually show the highest expression of caveolin 1, which is also found in the human BBB vessels (Dermeitzel and Krause, 1991; Laterra and Goldstein, 1993).

Interestingly, caveolin 1 has also been detected in the same embryonic area in rat embryos, including endothelial cells of blood vessels and the adjacent neuroectodermal cells, at an equivalent developmental stage (E12.7) (Parvas and Bueno, 2010a). Although few data are available on protein transport from blood plasma to eCSF in mammals at this developmental stage, the presence of caveolin 1 in the same region that involves both endothelial and neuroepithelial cells has led to the suggestion that there is a barrier function in mammals that acts in the same way as the function that has been demonstrated in chick embryos (Parvas and Bueno, 2010a).

### Traffic of water, ions and glucose occurs at the same transport site as proteins

During the initial stages of brain development, the control of water flux and regulation of solute transport is critical, since the increase in brain cavity volume is accompanied by a parallel increase in eCSF volume (reviewed by Gato and Desmond, 2009). Aquaporins (AQPs), a family of transmembrane proteins that form molecular water channels that mediate rapid membrane water transport, have been identified in epithelial and endothelial cells at numerous locations in higher vertebrates (Nielsen and Agre, 1995; Verkman et al., 1996; Agre et al., 1998, 2002). Of the 12 AQPs described to date (AQP0 to AQP11), AQP1 and AQP4 appear to play a role in water transport across brain barriers. AQP1 is expressed at very early stages of choroid plexus development in a range of mammalian species (Johansson et al., 2005), and AQP4 is present in adult brain endothelial cells (Amiry-Moghaddam et al., 2004). Findings from several studies suggest that choroid plexus apical expression of AQP1 is closely related to the rate of CSF formation. For example, AQP1-null mice have lowered CSF formation and pressure (Oshio et al., 2005).

Similarly, ion transfer appears to be critical for eCSF production and brain anlagen growth. Ions can move through cell membranes, both actively via ion pumps and passively via ion channels. In the adult, AQP4 co-localizes with the inwardly rectifying K^+^ channel Kir4.1 (Nagelhus et al., 2004), and it has been suggested that together, these two proteins play a key role in K^+^ and water balance. A study using PCR analysis and immunohistochemical procedures revealed that Kir4.1, AQP1, and AQP4 are present in both chick and rat embryos in the same blood vessels where specific protein transport has also been detected (Parvas and Bueno, 2010b; Figure 4). In chick embryos, AQP1 is present from E4, and AQP4, and Kir4.1 from E5; and in rat embryos AQP1 is detected from E12.7 and AQP4, and Kir4.1 from E13.7, i.e., at the equivalent developmental stages. These results may explain the control of the rapid expansion of brain anlagen at this developmental stage by means of water and ion influx, as previously suggested by Desmond and Jacobson (1977).

In addition to water and ion influx, the transfer of energy molecules such as glucose is crucial for the developing neuroepithelium. Glucose is an important energy source for the brain, and although few data are available on embryos, it has been recently shown that several glucose transporters are expressed at a much higher level in the rat embryonic choroid plexus than in the adult (Liddelow et al., 2014). Glucose is transported across cell membranes mainly by the GLUT family of solute carriers. In adults, GLUT1 is located in the brain barriers and also in the choroid plexus epithelial cells (Saunders et al., 2013). Similarly, a study using PCR analysis and immunohistochemical procedures demonstrated that in chick and rat embryos, GLUT1 is also present in the same blood vessels where protein transport and the presence of AQP1, AQP4, and Kir4.1 have been detected (Parvas and Bueno, 2010b).

## Conclusion

Taken together, all of these findings seem to confirm that a blood–eCSF barrier function controls eCSF composition and homeostasis from early stages of brain development in chick embryos. That control includes protein, glucose, water, and ion influx, and thus regulates eCSF osmolarity. It has been proposed that a similar blood–eCSF barrier is also present in mammals at the equivalent brain development stages (Parvas and Bueno, 2010a,b). The roles ascribed to this transient barrier confirm the crucial importance of the eCSF milieu for brain development at the beginning of primary neurogenesis; after the closure of the anterior neuropore and before the fetal choroid plexus is fully functional, which occurs shortly afterwards. Thus, the blood–eCSF barrier function should be considered as an actual barrier system that operates in early embryos in a transcellular way, and which includes specific transporters for particular molecules and sensors to monitor eCSF homeostasis. Just as most civilizations develop along riverbanks and seashores, using the fluid medium that is immediately available to them to promote cohesion and transport, and to enhance the chances of survival of the people who live at the edge of the liquid medium, so the brain is also organized, from its embryonic beginnings and throughout adult life, around an extraordinarily dynamic, and complex fluid: the CSF. Continuing this simile, as civilizations build harbors from which to ship goods and control transport that becomes ever more complex as they develop, so the brain has evolved barrier mechanisms, which start to form very early in brain development and change their morphology and physiology in accordance with the changing developmental stages.

### Conflict of interest statement

The authors declare that the research was conducted in the absence of any commercial or financial relationships that could be construed as a potential conflict of interest.

## Abbreviations

BBB: blood–brain barrier
CG: Carnegie Stage (for human embryo development)
CNS: central nervous system
CSF: cerebrospinal fluid
E: embryonic developmental day
eCSF: embryonic cerebrospinal fluid
EGF: epidermal growth factor ESI-MS/MS: electrospray ionization coupled with tandem mass spectrometry
fCSF: fetal cerebrospinal fluid
FGF2: fibroblast growth factor no. 2
HRP: horse radish peroxidase
IsO: mesencephalic-rhombencephalic isthmus organizing center
RBP: retinol binding protein
SDS-PAGE: sodium dodecyl sulfate polyacrylamide gel electrophoresis.
